# Hydrogen Sulfide and Carbon Monoxide Tolerance in Bacteria

**DOI:** 10.3390/antiox10050729

**Published:** 2021-05-05

**Authors:** Sofia S. Mendes, Vanessa Miranda, Lígia M. Saraiva

**Affiliations:** Instituto de Tecnologia Química e Biológica António Xavier, Universidade Nova de Lisboa, Avenida da República, 2780-157 Oeiras, Portugal; ass.mendes@itqb.unl.pt (S.S.M.); vmiranda@itqb.unl.pt (V.M.)

**Keywords:** bacteria, hydrogen sulfide, carbon monoxide, CORMs

## Abstract

Hydrogen sulfide and carbon monoxide share the ability to be beneficial or harmful molecules depending on the concentrations to which organisms are exposed. Interestingly, humans and some bacteria produce small amounts of these compounds. Since several publications have summarized the recent knowledge of its effects in humans, here we have chosen to focus on the role of H_2_S and CO on microbial physiology. We briefly review the current knowledge on how bacteria produce and use H_2_S and CO. We address their potential antimicrobial properties when used at higher concentrations, and describe how microbial systems detect and survive toxic levels of H_2_S and CO. Finally, we highlight their antimicrobial properties against human pathogens when endogenously produced by the host and when released by external chemical donors.

## 1. Introduction

Hydrogen sulfide (H_2_S) and carbon monoxide (CO) are small molecules that are historically related with environmental industrial pollution. However, the two compounds have long been recognized as also being produced by mammalian and bacterial cells in low amounts that mediate important physiological processes [1,2]. 

H_2_S diffuses through cell membranes and inside cells, at physiological pH, is mainly present in the deprotonated conjugate base form of hydrosulfide anion (HS^−^). Here, we will use sulfide to refer collectively to the H_2_S and HS^-^ forms. In organisms, H_2_S is the product of enzymes of the trans-sulfuration pathway. Mammals express three H_2_S generating enzymes: cystathionine β-synthase (CBS), cystathionine γ-lyase (CSE), and 3-mercaptopyruvate sulfurtransferase (3MST or MPST). CBS and CSE form H_2_S predominantly from l-cysteine, while the 3MST enzyme generates H_2_S via the synthesis of the intermediate 3-mercaptopyruvate, which is produced by cysteine aminotransferase [3,4,5]. 

Carbon monoxide present in the environment is a product of fuel combustion. Mammals also produce CO endogenously through heme oxygenase (HO) enzymes that degrade heme releasing CO, biliverdin, which is reduced to bilirubin and ferrous iron, which is scavenged by ferritin. Humans express three HO isoforms: HO-1, which is inducible by heme and oxidative stress; HO-2, which is constitutively produced; and HO-3, which is a poor heme degrading catalyst and is most likely a regulator of proteins, such as HO-1. The products of HO-catalyzed reactions are considered to play a relevant role in oxidative stress protection of cells [6]. CO has potential for therapeutic applications through three modes of delivery: induction of genes encoding heme oxygenases; inhalation of gaseous CO; and use of CO-releasing molecules (CORMs) [7]. CORMs exhibit vasodilatory, regulation of mitochondrial respiration, anti-inflammatory, anti-apoptotic, anti-ischemic, and cardioprotective properties [8,9].

Prokaryotes also utilize and generate H_2_S and CO, and their important role may be inferred from the widespread presence in the microbial genomes of putative orthologs, of at least one, of the eukaryotic H_2_S and CO producing enzymes. At physiological pH, the lifetimes of H_2_S and CO are quite different: while H_2_S has a reduced lifetime, the greater stability of CO allows it to have effects in sites distant from where it is produced. The beneficial or harmful effects of H_2_S and CO depend mainly on their concentrations, but also on the organisms and environmental conditions. At high concentrations, both are toxic to mammals and microbes, and, more recently, their antimicrobial potentials have been explored. This review summarizes the current knowledge on the antimicrobial properties of H_2_S and CO and the physiological adaptation of microbes when exposed to these stresses. 

## 2. Bacterial Responses to H_2_S

In general, low concentrations of H_2_S in the micromolar range are cytoprotective, but millimolar concentrations are cytotoxic to microbes, and some prokaryotes contain proteins for protection against H_2_S efflux transporters [10,11,12].

High concentrations of sulfides hamper bacterial growth, as shown for *Escherichia coli, Shewanella oneidensis, Aspergillus niger*, *Penicillium italicum,* and *Acinetobacter baumanni.* In all these microorganisms, sulfide inhibits the activity of superoxide dismutase and catalase enzymes that are linked to cellular defenses against oxidative stress. Consequently, it causes an elevation of the intracellular reactive oxygen species (ROS) content and reduction of the glutathione levels [13,14,15]. In *A. baumanni*, NaHS (80–160 µM) caused membrane depolarization and lowered the ATP levels [16]. In general, the toxicity of sulfide, besides being associated with oxidative damage via inhibition of antioxidant proteins, also results from DNA damage, lipid peroxidation, protein denaturation through disulfide disruption, and inactivation of redox centers in metalloenzymes due to its binding to the metals [1,17,18]. 

In addition, toxic sulfur-containing compounds produced by bacteria contribute to antagonistic interactions with microbes occupying the same niche [19]. *Proteus mirabilis*, member of the Enterobacteriaceae family and a H_2_S producer, exerts bactericidal effect over *E. coli*, *Klebsiella pneumoniae* and *Morganella morganii* in planktonic cells and mixed biofilms [20].

**Figure 1:**
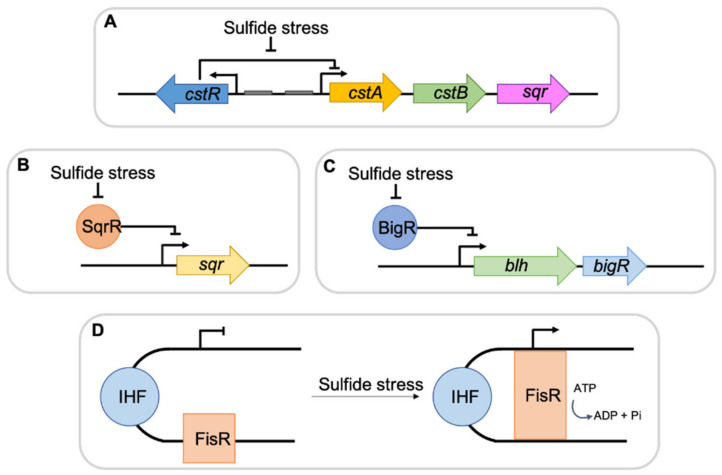
General scheme summarizing bacterial H_2_S regulators. (**A**) CstR regulator binds to the upstream region of the *cst* genes repressing their expression. During sulfide stress, the DNA-binding affinity of the repressor decreases allowing for RNA polymerase binding and gene transcription. (**B**) and (**C**) SqrR and BigR bind to promoter regions of *sqr*, *blh* and *bigR* genes, inhibiting transcription. In the presence of sulfide, the repression is lifted and genes expression occurs. (**D**) Under sulfide stress, FisR hydrolizes ATP to ADP plus free phosphate, resulting in RNA polymerase activation and increase in gene expression.

Several sulfide-specific transcription repressors, such as CstR, SqrR/BigR, FisR and CsoR have been identified [21,22,23] and, in *E. coli*, sulfide was also described to activate two major redox-responsive transcriptional regulators, namely SoxRS and OxyR [13] (Figure 1).

How bacteria respond to sulfide toxicity has been addressed by a limited number of works, which include transcriptomic and proteomic studies. Analysis of the transcriptome of *A. baumanni* when exposed to sulfide stress generated by 0.2 mM Na_2_S [22], revealed the up-regulation of genes encoding persulfide dioxygenase (PDO1), sulfide:quinone oxidoreductase (SQR), a putative sulfite effluxer TauE, terminal ubiquinol oxidase of the cytochrome *bd*-type, an oxidase that is resistant to H_2_S [24], a putative copper transport OprC, ferritin-like gene products, flavohemoglobin/nitric oxide dioxygenase, and the [4Fe-4S]-containing nitric oxide-sensing transcriptional repressor (NsrR). Genes with reduced expression included those related to uptake of sulfur and derivatives, such as the ABC transporters putatively involved in the uptake of sulfonate taurine and inorganic sulfate, and putative glutamate and aspartate transporters. Interestingly, only a few members of the OxyR regulon were modified, indicating that, in this pathogen, OxyR does not respond directly to sulfide, and suggesting that the responses to Na_2_S and H_2_O_2_ stress differ significantly. Concerning the proteomic data [24], exposure of *A. baumanni* to sulfide increased the abundance of ROS-detoxification enzymes (heme-catalase, superoxide dismutase, alkyl hydroperoxidase, and universal stress proteins), metabolic enzymes (aconitase, isocitrate lyase, succinate semialdehyde dehydrogenase, and malate synthase), and proteins that respond to high-Fe and high-Cu levels, such as the periplasmic and cytoplasmic copper chaperones (CusF, CopZ), lipoprotein NlpE, and the iron storage ferritin FntA and bacterioferritin, of which corresponding genes were also seen induced in the transcriptome analysis. Furthermore, cells lacking the FisR regulator and that were exposed to H_2_S showed elevated abundance of a ferric siderophore receptor protein, a glutathione-dependent disulfide bond oxidoreductase, and a nitrite/sulfite reductase.

In *Bacillus subtilis* and *Staphylococcus aureus*, high amounts of sulfide repressed the transcription of cysteine synthase (*cysK*), *cysM* encoding CBS, and *metB* encoding CSE, which allow sulfur assimilation from thiol and homocysteine. Also repressed were the genes coding for methionine and cysteine ABC transporters, the operon for a sulfurtransferase-like protein, and a gene of a putative thiosulfate importer [25,26,27]. In *S. aureus*, exogenous sulfide induced the copper-sensing *cst* operon that is mediated by the sulfurtransferase repressor CstR. Cst includes proteins that mitigate sulfide toxicity, such as CstA and CstB, that are a multidomain sulfurtransferase and a non-heme Fe persulfide dioxygenase, respectively, and the SQR sulfide:quinone oxidoreductase that catalyzes the oxidation of sulfide to sulfane sulfur [28]. Consistent with the data, strains deleted in the genes of the *cst* operon (Δ*cstA*, Δ*cstB*, and Δ*sqr*) exhibited impaired growth in the presence of NaHS. Sulfide also modifies genes encoding enzymes and regulators involved in sugar (*glpF*, *marR*, *gapB*, *scrR*, *gntK*, and *gntR*) and amino acid (*putA*) metabolisms [29]. The overall transcription pattern suggests that the *S. aureus* response to sulfide shares similarities with that under zinc limitation. For example, sulfide upregulates the zinc uptake repressor (Zur) regulon, represses zinc transporters and a zinc-binding lipoprotein. It induces genes for manganese transporters MntABC, that are controlled by the MntR repressor, and the Co/Ni uptake system. Accordingly, sulfide decreases the intracellular Zn levels by approximately 10-fold, causing only a small reduction of the intracellular Cu/Ni levels, and no alteration in Mn/Fe levels [30].

## 3. H_2_S Producing Bacteria Confer Self-Protection against Oxidative Stress

Bacteria can produce sulfide as by-product of its sulfur metabolism, e.g., the intestinal sulfate-reducing bacteria (SRB), and through cysteine desulfurases. In general, sulfide releasing bacteria support quite high concentrations of sulfide, as is the case of the SRB *Desulfovibrio piger* that grows in concentrations up to 4 mM of sulfide [29]. However, the species that co-live in the gut environment, such as *Lactobacillus* spp., are affected in a way that varies among species. For example, sulfide is more toxic to *L. pentosus*, *L. paracasei* and *L. reuteri* than to *L. fermentum* and *L. plantarum* [30].

A large number of bacteria contain at least one ortholog of the eukaryotic H_2_S producing enzymes, namely CBS, CSE or 3MST. In bacteria, as well as in eukaryotes and plants, endogenous sulfide production has been shown to be an important protective mechanism against oxidative stress and antibiotics. Specifically, inactivation of *cbs*, *cse* and *3mst* genes in *B. anthracis*, *Pseudomonas aeruginosa*, *S. aureus*, *E. coli* and *Mycobacterium tuberculosis* resulted in strains less resistant to oxidative stress [11]. The ability of sulfide to mitigate oxidative stress stems apparently from various related factors. Sulfide promotes reduction of intracellular levels of cysteine, mediates sequestration of free iron reducing oxidative stress derived from the Fenton reaction, and induces genes encoding antioxidant enzymes. In these processes, the iron uptake regulator Fur appears to play a role, as shown for an *E. coli* strain Δ*fur* Δ*3-mst* mutant strain that had enhanced susceptibility to ROS. Consistent with these data, overexpression of *3-mst* in *E. coli* Δ*fur* exhibited reduced DNA damage and decreased cell death. Furthermore, under oxidative stress, the up-regulation of *E. coli 3-mst* is also triggered by the CysB regulator, that controls the transcription of several genes related to sulfur metabolism including the cysteine importer TcyP. Depletion of cysteine levels that occurs during oxidative stress activates the CysB regulon. The consequent induction of TcyP increases the influx of cysteine/cysteine thus resulting in elevated expression of 3-MST [31,32]. 

Nonetheless, more studies are required to allow for generalization of H_2_S as a ROS protector molecule. For example, in *S. oneidensis*, the protective effect only occurred when H_2_S was applied to cells prior to exposure to the oxidative stress effector (H_2_O_2_), while the simultaneous addition of H_2_S and H_2_O_2_ caused cell growth inhibition. The mechanism is still unclear, however authors have proposed that sulfide protection to *Shewanella* spp. could have physiological relevance as the bacterium resides in iron and sulfur rich niches [14]. 

## 4. H_2_S and Microbial Antibiotic Resistance

A new mode of antibiotic resistance mediated by sulfide was reported in pathogenic bacteria that involves inhibition of the oxidative stress imposed by ROS-generating antibiotics. Several studies described that the genes encoding H_2_S-releasing enzymes, such as CBS and CSE, in *B. anthracis*, *P. aeruginosa*, *S. aureus*, and *M. tuberculosis,* or 3-MST in *E. coli*, contribute to tolerance to gentamicin, ampicillin and nalidixic acid [31,33]. 

In *E. coli* and *M. tuberculosis*, supplementation of cysteine or other small thiols also increased the resistance to gentamicin and rifampicin, respectively [34,35]. In *E. coli*, treatment of cells with ampicillin augmented the levels of cytochrome *bo*_3_ oxidase (*cyoA*) and lowered the expression of the cytochrome *bd* quinol oxidase (*cydB*). However, pre-exposure of cells to sulfide reversed the expression pattern, and the more sulfide-resistant cytochrome *bd* oxidase became prevalent. Moreover, sulfide protected *cyoA* mutant from ampicillin toxicity but was ineffective in protecting the *cydB* mutant. Thus, the presence of sulfide forced *E. coli* to continue respiration catalyzed by cytochrome *bd*. Although less efficient, this alternative respiratory pathway based on a sulfide-resistant enzyme, which also appears to be able to act as catalase and quinol peroxidase, enhances the bacteria resistance to antibiotics [24,35]. 

Still, more recent studies have called into question the widely held notion that sulfide is a bacterial defense mechanism against antibiotics. In *S. aureus*, sulfide exacerbated the killing by antibiotics such as quinolones, and the sulfide-mediated protection was limited to aminoglycosides, such as gentamicin. Furthermore, the sulfide-induced tolerance to gentamicin was due to the decrease in gentamicin uptake and not to the reduction of oxidative stress [31]. 

In *A. baumanni*, which does not produce sulfide endogenously, co-treatment of antibioticsand NaHS potentiated the activity of ROS-producing antibiotics such as gentamicin, colistin, rifampicin and clarithromycin by several orders of magnitude. In this case, the effect of sulfide, which is opposite to what would have been expected from the results described above for *E. coli*, seems to be linked with the ability of H_2_S alone to compromise bacterial cell redox homeostasis [16]. Nevertheless, the results open a not yet tested possibility that sulfide could be used per se and in combination with antibiotics as antimicrobials against drug resistance of non-sulfide producing pathogens. 

## 5. H_2_S in Host-Pathogen Interactions

Several works indicate that upregulation of genes encoding bacterial enzymes involved in sulfide biogenesis is an important adaptive response of pathogens during the infection process. Host-generated sulfide seems to modulate the course of bacterial and viral infections as H_2_S activates macrophages and the phagolysosomal fusion process, resulting in significant enhancement of phagocytosis. Sulfide triggers induction of endogenous mammalian antioxidant defenses protecting cells from infection-associated oxidative stress [36,37]. It also inhibits the inflammatory response by suppressing the endotoxin-induced tumor necrosis factor a (TNFα) produced by macrophages [31,37,38,39].

Lipopolysaccharide (LPS), the cell wall component of Gram-negative bacteria that has inflammatory properties, stimulates sulfide production in human macrophages via NF- κB /ERK [40]. In mouse models of septic shock, LPS raised the CSE expression in liver and kidney resulting in augmented levels of H_2_S in tissues and serum [41,42]. In an animal model of sepsis, induced by *Streptococcus pneumonia*, infusion of NaHS reduced the sepsis–related lung, kidney injury and distant organ injury without apparent bacterial outgrowth [43]. 

Interestingly, it was reported that host-derived H_2_S protects against viral infections, including COVID-19, by mechanisms that involved modulation of the NF-kB signaling [35,44,45,46]. 

In *M. smegmatis* viability in macrophages is enhanced in hosts with blocked trans-sulfuration pathway, while treatment with *N*-acetylcysteine, that augments the cysteine flux through the sulfide pathway, potentiate bacteria killing. A similar effect was observed in *Mycoplasma fermentans* infected macrophages, in which the mammalian cells derived sulfide reduces the inflammatory response through a mechanism that involves inhibition of NF-κB activation and nuclear translocation, and consequent decrease of the transcription of pro-inflammatory genes and of pro-inflammatory cytokines production. In Mycoplasma infected macrophages, H_2_S upregulated the Nrf2/HO-1 pathway activating downstream HO-1 and superoxide dismutase 1 (SOD1), thus reducing intracellular ROS levels [37,47,48,49,50].

On the contrary, an *M. tuberculosis* infected host that actively produces sulfide seems to have an aggravated course of the infection. Low concentrations of a slow sulfide releaser increased the levels of glycolytic and TCA cycle metabolic intermediates, and promoted oxygen respiration at the level of the cytochrome *bd* quinol oxidase, altogether stimulating *M. tuberculosis* growth. In addition, a transcriptomic analysis revealed the upregulation of genes belonging to the DosR/S/T dormancy regulon, and CsoR and RicR copper regulons suggesting that sulfide triggers *M. tuberculosis* dormancy [51]. Additionally, *M. tuberculosis*-infected mice that produce H_2_S exhibited an excessive innate immune response, with suppression of the adaptive immune response, decreased levels of cytokines, such as IL-1β, IL-6, IL-9, IL-12, TNF-α, IL-17, IFN-γ, and inhibition of the central carbon metabolism. Consistent with this, infected animals with no capacity to produce H_2_S survived longer, had lower bacterial burden in the lungs, spleens, and livers, and impairment of the central carbon metabolism was not observed. Thus, the excessive amount of sulfide produced by *M. tuberculosis*-infected macrophages and the lower amounts of pro-inflammatory cytokines circulating in the animal promote *Mycobacterium* spp. growth, and consequently the exacerbation of the tuberculosis infection [34].

On the other hand, inhibition of sulfide producing enzymes in *E. coli* and *S. aureus*, chemically or by gene deletion, lowered the bacterial loads in leukocytes and macrophages. When compared with the wild type, the sulfide-deficient strains are less resistant in infected mice, with animals having lower bacterial burden and IL-6 levels in the spleen and plasma, respectively [52,53,54].

Hydrogen sulfide has been implicated in ulcerative colitis and to contribute to halitosis, both conditions related with hosts with proliferation of anaerobic bacteria such as SRB and periodontopathogenic bacteria, respectively [52,54].

## 6. CO Utilizing Bacteria

Carbon monoxide is utilized by several bacteria as energy source, e.g., *Rhodopseudomonas* sp., *Methanosarcina barkeri* and *Methanobacterium formicicum* [55]. In the dark, *Rhodospirillum rubrum* uses CO-H_2_ as an energy source, reaching growth rates of approximately 80% when compared with light-driven growth. *Clostridium ljungdahlii* and *C. autoethanogenumis* also use CO as a carbon source, producing ethanol in the process [56,57]. The CO oxidation systems are spread in the microbial world [55,58,59,60], and present in *Carboxydothermus hydrogenoformans* [61], *Azotobacter vinelandii* [62], *Mycobacterium* spp. [63], and in some sulfate reducing bacteria, including the thermophilic archaeon *Archaeoglobus fulgidus* in which CO is an electron donor for sulfate reduction. The CO tolerance of these strains is considered a beneficial alternative to biodesulfurisation processes [64,65,66].

Growth on CO is sustained by carbon monoxide dehydrogenase (CODH) enzymes that catalyze oxidation of CO to CO_2_, that is transformed into cellular carbon by reductive CO_2_ fixation pathways, such as the Calvin–Benson–Bassham cycle, the reverse tricarboxylic acid cycle, the 3-hydropropionate cycle or the Wood–Ljunddahl pathway [59]. The processes coupled to CO oxidation are oxygen respiration, hydrogenogenesis, sulfate or sulfur respiration and carbonate respiration [67]. In several bacteria, CODH enzymes are encoded by the *cox* operon that is composed of *coxS*, *coxM* and *coxL* genes expressing an iron-sulfur protein, a flavin adenine dinucleotide-binding protein and a catalytic molybdenum cytosine dinucleotide-binding protein, respectively. In agreement, strains inactivated in CODH encoding genes cannot use CO as electron acceptor [64,68]. 

Transcriptomics studies of CO-oxidizing bacteria in the presence of CO gas were done for *A. fulgidus,* *Parageobacillus thermoglucosidasius*, *Calderihabitans maritimus* and *C. pertinax*. The general trend was the upregulation of CODH encoding genes, and in *P. thermoglucosidasius* a transcriptional pattern related with transition from aerobic to anaerobic growth was observed [56,65,66,67].

## 7. Bacterial Responses to Toxic CO

CO produces no major effects on humans when inhaled at very low concentrations and/or for a short period of time. However, high doses and prolonged exposure may cause symptoms such as visual disturbances and seizures and, when in concentrations above 2000 ppm can induce coma or even death. In mammals, CO binds to hemoglobin, having approximately 250 times more affinity to the protein than oxygen, leading to the formation of carboxyhemoglobin and reducing the oxygen carrying capacity of the blood, causing tissue hypoxia [8,69]. High levels of CO in the blood is associated with aggravated asthma, cystic fibrosis, diabetes, cardiac disease and severe renal failure [70,71]. 

High concentrations of CO also have an inhibitory effect on bacteria, and CO gas and CO-releasing molecules (CORMs) show antimicrobial properties in the micromolar range of concentrations, as first reported for *E. coli* and *S. aureus*, grown under aerobic and anaerobic conditions [72], and later for several other pathogens [73]. CORMs are, in general, organometallic complexes that release CO intracellularly in a controlled and efficient way and reaching concentrations higher than CO gas, whose solubility is low [69,74]. CORMs are considered as non-toxic to eukaryotic cells and mice [75,76]. CORMs may have an additive effect when combined with other antibiotics as shown for *Helicobacter pylori* and *P. aeruginosa*. Furthermore, in *H. pylori*, CORMs contributed to overcoming antibiotic resistance of clinical isolates [77,78,79]. Exposure of *E. coli* and *P. aeruginosa* to CO releasers also prevented biofilm maturation and killing of bacteria within an established biofilm [79,80]. A summary of the currently available data on the antimicrobials properties of several CORMs tested against a wide range of pathogens is presented in Table 1. 

Studies on the bactericidal mode of action of CO done in *E. coli*, *P. aeruginosa*, *H. pylori* and *Campylobacter jejuni* showed that CO decreases the respiratory rates due to the direct binding to terminal oxidases [77,78,85,96]. Moreover, bacterial cells treated with CORM contained high intracellular ROS level [97,98,99]. Still, CO also targets non-heme proteins, as inferred by the similar CO susceptibility of heme-deficient (Δ*hemA*) and wild-type strains of *E. coli* [100]. A metabolomic study of *E. coli* treated with the CO releaser CORM-3 reported the impairment of glutamate synthesis and inactivation of iron-sulfur enzymes, such as aconitase and fumarase, causing intracellular glutamate deficiency and inhibition of the nitrogen and TCA cycles [101]. In strains of sulfate-reducing bacteria of the *Desulfovibrio* genus, high CO concentrations (20–70% *v/v*) inactivated hydrogenase and superoxide dismutase enzymes, and stimulated formation of ROS [87,88,89]. CO interacts with proteins such as albumin, ferritin and lysozyme via a protein-Ru(II)-(CO)_2_ adduct. The formation of this complex accelerates the release of CO from CORM-3, suggesting that plasma proteins may control the pharmacokinetic properties of CORMs [102]. Moreover, CO maintains its bactericidal properties under anaerobic conditions, and the absence of oxygen may even increase its toxicity as shown for *E. coli* and *S. aureus* [72]. Thus, in addition to its direct ligation to iron, other intracellular CO targets remain to be identified due to the affinity tometal atoms, such as cobalt, nickel and copper [103,104].

**Figure 2:**
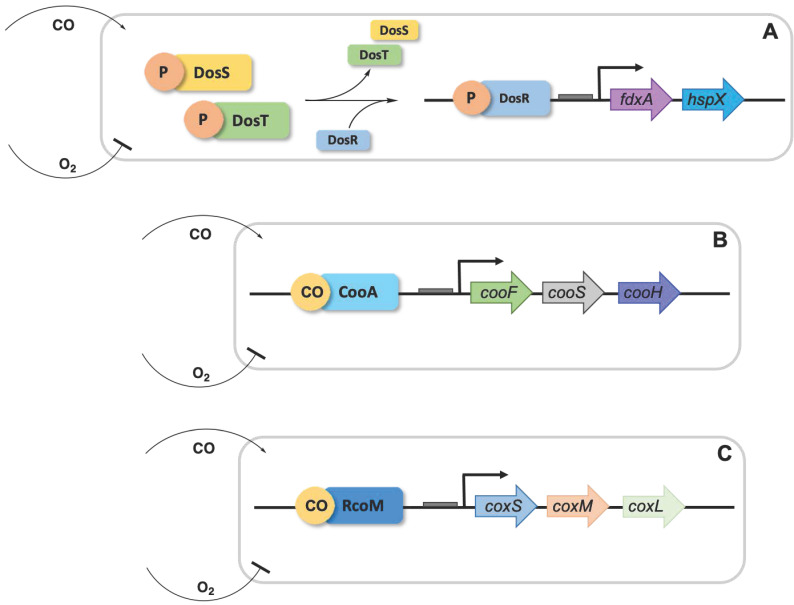
General scheme on the action of CO regulators. (**A**) Upregulation of the dormancy of DosR-phosphate transcriptional regulator is triggered by CO and H_2_S. (**B**) and (**C**) The CooA and RcoM regulators, which are activated by CO, upregulate the transcription of the *coo* and *cox* gene products that catalyze conversion of CO to CO_2_.

Bacteria rely on CO sensors and CO-dependent regulators to utilize or control intracellular CO levels, most of them heme-containing proteins (Figure 2). However, CO has the ability to displace histidine, cysteine and tyrosine residues that are coordinated to metals. Thus, in several proteins, the displacement by CO of the proximal ligand of heme iron histidine is the basis of sensor functioning [105].

One of the best studied CO-regulators is CooA that is a member of the FNR/CRP family of transcriptional regulators and is present in a wide variety of bacteria. CooA is a homodimeric protein that upon CO binding to the heme undergoes a conformational change that triggers DNA ligation to the *coo* promoter, regulating the CO oxidation system [7,106]. 

Some bacteria contain another type of CO regulator, namely RcoM, that upon binding of CO to its heme moiety controls transcription of *coo* and *cox* genes [107,108]. CooA responds only to CO, but other heme-based CO sensors also bind oxygen, namely *Sinorhizobium meliloti FixLJ*, *Acetobacter xylinum* AxPDEA1, *B. subtilis* HemAT, and *E. coli* Dos [109,110,111,112]. 

In *M. tuberculosis*, the kinases DosS (also known as DevS) and DosT are linked to dormancy. At high concentrations, CO binds to their heme groups promoting autophosphorylation and the subsequent phosphorylation of the DosR dormancy regulator leads to induction of the dormancy operon [113,114].

Several works on the bacterial response to the stress imposed by CO and CORMs have been published. A transcriptomic study of *E. coli* exposed to CO gas revealed changes in the expression pattern of ~30% of the whole genome [115]. In aerobic-grown *E. coli* cells, CO caused downregulation of several TCA cycle related genes, increase expression of *cydAB* encoding cytochrome *bd-I*, and upregulation of NADH dehydrogenase (*ndh*). Furthermore, CO mimicked anaerobic conditions as judged by the down-regulation of some of the ArcA regulated genes involved in the tricarboxylic acid cycle (TCA). These data were interpreted as resulting from the direct competition of CO for the oxygen binding sites of ArcA, which is a regulator that mediates the transcription of ~11 operons during an aerobic-anaerobic transition. CO gas also modified the expression of genes regulated by FNR, a major bacterial transcription factor that contains an [4Fe-4S] oxygen sensor cluster and represses over 100 genes, raising the possibility that it could be inactivated through CO binding to the iron–sulfur cluster. Additionally, the elevated expression of genes involved in arginine, taurine, and methionine biosynthesis, iron acquisition, and sulfur utilization/uptake, suggests that, in response to CO, cells undergo a shortage of amino acids, sulfur, and iron [115].

*E. coli* exposed to CO donors, such as CORM-2 and CORM-3 also caused major alteration of the mRNA abundance of a large number of genes [100,116]. In general, it resulted in the down-regulation of genes involved in the citric acid cycle and respiration (*cyoABCDE* and *sdhABCD* operon). However, small induction of the *cydAB* genes encoding cytochrome *bd*-I was observed, which is consistent with cytochrome *bd*-I being the more CO resistant oxidase [117]. Up-regulated genes were those participating in SOS response, DNA repair, protein homeostasis, zinc, methionine, sulfur and cysteine metabolism, and biofilm formation [85,116]. In general, the transcriptome alteration shared similarities with those observed for *E. coli* under oxidative stress (e.g., induction of members of the SoxRS regulon). Interestingly, in uropathogenic *E. coli* isolates, CORM-2 induced up-regulation of some virulence genes [81,82].

## 8. CO Producing Bacteria

Several pathogens, like *S. mitis* and *B. cereus*, cause hemolysis during infection [118], and use heme-oxygenase enzymes to obtain iron from heme degradation. The first described bacterial heme oxygenase was HmuO of *Corynebacterium diphtheriae*. Over the years, several other heme oxygenases, regulated by intracellular iron concentration, have been found in pathogens, such as Cj1613c in *C. jejuni*, HugZ in *H. pylori*, and HemO in *Neisseria meningitides* [113,119,120,121,122,123,124].

Of note, some bacterial heme oxygenases release formaldehyde instead of CO, as for example the *S. aureus* monooxygenases IsdG and IsdI, and *M. tuberculosis* MhuD [125]. In *S. aureus*, IsdG was proposed to link biosynthesis and uptake heme pathways, thus protecting the bacteria from intracellular heme toxic accumulation [126,127]. 

As mentioned above, endogenously produced sulfide appears to protect bacteria from a broad range of antibiotics. However, no similar effect was, so far, observed with CO, since CO gas only marginally improved *E. coli* growth in the presence of the antibiotics such as doxycycline, trimethoprim or cefotaxime [115]. 

## 9. CO and H_2_S in Host-Pathogen Interactions

The finding that HO-1 is highly induced in macrophages in response to bacterial infections, and that animals with blocked HO-1 were highly sensitive to bacteria and presented signs of systemic inflammatory response led to the proposal that CO enhances bacterial clearance [128]. In addition, HO-1-deficient mice showed increased bacteremia and lethality during sepsis, and their survival improved upon administration of CO donor molecules [73,129]. Moreover, Morse and colleagues reported that CO gas inhalation increased the survival of a sepsis mice model and reduced the levels of pro-inflammatory cytokines, and that overexpression of HO-1 in macrophages caused decrease in the cytokine levels [130]. In another study, infections of the upper respiratory tract by the influenza virus elevated the amount of CO exhaled by patients, which was considered to be derived from the induction of heme oxygenase in macrophages and epithelial cells [123]. Pamplona et al. reported that inhalation of CO gas protected *Plasmodium berghei* infected mice from development of cerebral malaria, although it did not decrease parasitemia. Authors proposed that binding of CO to heme prevents the disruption of the blood–brain barrier and the consequent development of cerebral malaria associated to high toxic levels of free heme in erythrocytes derived from hemolysis [131,132].

However, some bacteria seem to be able profit from induction of host-derived CO production as a self-protection mechanism, as was shown for *M. tuberculosis* that induced HO-1 in infected macrophages with release of CO that triggers the dormant stage of *M. tuberculosis* [133].

## 10. H_2_S and CO Interplay

The chemistry and biology of H_2_S and CO are interconnected due to the shared capacity of these molecules to interact with metal centers and cysteine residues in proteins. Both molecules are involved in related signaling pathways, in which they promote activation/inactivation of the target proteins and may interfere in the level and activity of each other. For example, the H_2_S-generating CBS enzyme is reversibly inhibited by the CO derived from heme oxygenase, which is proposed to have pathophysiological implications including on the metabolism of cancer cells. Theregulatory heme-binding domain of the human CBS is absent from the homolog enzymes from prokaryotes or unicellular eukaryotes [134].

H_2_S was reported to increase the expression of heme oxygenase, through the action of the Keap1/Nrf2 system on the HO-1 enzyme and because the intracellular sulfide levels seem to modulate the HO-2 activity through the several Cys-Pro signatures present in HO-2 that regulate heme binding [135]. Moreover, H_2_S is proposed to react with ferric verdoheme, an intermediate of the HO-2 activity, thus modifying its oxygen-sensing activation mode [136]. Accordingly, H_2_S was shown to upregulate the heme oxygenase in the pulmonary arteries of hypoxic rats and stimulate heme oxygenase levels in mouse retinal ganglion cells [137].

As mentioned above, both CO and H_2_S deplete bacterial intracellular zinc levels and that in *M. tuberculosis* the two molecules induce the dormancy phase. Although several prokaryotes encode simultaneously in their genome enzymes that produce CO and H_2_S, the relationship between the function of the two molecules in bacterial physiology remains to be addressed. 

## 11. Conclusions

Along with nitric oxide, H_2_S and CO are double-edged molecules—if on the one hand are essential signaling molecules to human cells, on the other hand they can cause death. This duplicity of function, which took decades to be recognized, is reminiscent of what also happens with metals. As with these, everything depends on the concentration, which when high is toxic but in small amounts is beneficial and even essential for the normal maintenance of organisms.

More recently, it has been shown that CO and H_2_S can function as antimicrobials. There are already several examples of the effectiveness of CORMs as antimicrobials (Table 1), but similar studies remain to be done with H_2_S. In fact, sulfide and CO-based compounds may represent a novel kind of antimicrobials, with modes of action and targets that are different from those of the currently available antibiotics. And new antimicrobial drugs are urgently needed due to the growing number of infections caused by antibiotic-resistant pathogenic strains.

## Figures and Tables

**Table 1:** CORMs used as antimicrobials.

CORM	Organism	Concentration (µM)	Atmosphere	Observations
CORM-2	*H. pylori* [77]	195–390	Microaerobic	Parental strain (26695) and six clinical isolates (5599, 5611, 5846, 4597, 4574 and 5587)
	*E. coli* ATCC 23716 [72]	250	Anaerobic, aerobic	---
	*E. coli* MG1655 [76]	350	Microaerobic	---
	*E. coli* MG1655 [81]	500	Aerobic	---
	*E. coli* UPEC J96 [81]	500	Aerobic	---
	*E. coli* ESBL 7 [81]	500	Aerobic	ESBL clinical isolate 7
	*E. coli* ESBL 1, *E. coli* UPEC 2, *E. coli* MG1655 TG1 [80]	500	Aerobic	ESBL and non-producing (UPEC) UPEC isolates
	*E. coli* ESBL7, *E. coli* UPEC2, *E. coli* K12 [82]	500	Aerobic	ESBL-producing ESBL and non-producing UPEC isolates
	*S. aureus* NCTC8325 [72]	250	Aerobic, microaerobic	---
	*P. aeruginosa* [83]	10	Aerobic	---
	*P. aeruginosa* PAO1 biofilms [79]	25–200	Microaerobic	Static growth wells
CORM-3	*E. coli* MG1655 [84]	100	Aerobic	25% air saturation
	*E. coli* ATCC 23716 [72]	200–400	Anaerobic	---
	*E. coli* MG1655 [85]	30–400 100–200	Aerobic Anaerobic	---
	*S. aureus* NCTC8325 [72]	400	Microaerobic	---
	*P. aeruginosa* PAO1 ATCC 15692 [78,83]	10, 500	Aerobic	---
	*S. typhimurium* ATCC 14028s [86]	150	n/a	---
ALF850	*E. coli* MG1655 [76]	650	Microaerobic	---
ALF021	*E. coli* K12 ATCC 23716 [72]	200	Anaerobic	
	*S. aureus* NCTC 8325 [72]	500	Aerobic	
		600	Microaerobic	
TryptoCORM	*E. coli* W3110 [87,88]	100	Aerobic	With irradiation
	*N. gonorrhoeae* MS11 [88]	100	5% CO_2_	In the dark
	*S. aureus* 8325-4 [88]	100	Aerobic	With irradiation and in the dark
PhotoCORM	*E. coli* EC958 [89]	350	Aerobic	Pre-exposed to UV light
USC-CN028-31				
(Mn(CO)3(tpa-k3N)Br)				
	Avian pathogenic *E. coli* [90]	2000	Microaerobic	---
	*E. coli* K12 MG1655 [91]	250–500	Aerobic	Glucose or succinate as carbon source; Transient to severe reduction of growth
[Mn(CO)3(bpy)(mcz)]PF6	*S. aureus*, *S. epidermidis*	1.25	n/a	---
	*E. faecium*,			
	*L. major*,	1.8	n/a	---
	*T. brucei* [92]	0.4	n/a	---
[Mn(CO)3(bpy)(ktz)]PF6	*S. aureus*, *S. epidermidis*,	2.5	n/a	---
	*L. major*,	2	n/a	---
	*T. brucei* [92]	0.7	n/a	---
[Mn(CO)3(bpy)(ctz)]PF6	*S. aureus*, *S. epidermidis*,	0.6	n/a	---
	*E. faecium*, *E. faecalis*	2.5	n/a	---
	*L. major*,	2.2	n/a	---
	*T. brucei* [92]	0.5	n/a	---
CORM-371	*P. aeruginosa* [83]	10	Aerobic	---
CORM-1 nonwoven	*S. aureus* MRSA (biofilms) [93]	< 3 µmol CO/mg nonwoven	n/a	70% inhibition after irradiation at 405 nm
EBOR-CORM-1	*P. aeruginosa* [94]	500	Microaerobic	Tested in planktonic and biofilms of PAO1
CORM-401	*E. coli* K12 [95]	500	n/a	---
ALF062	*E. coli* ATCC 23716 [72]	50	Aerobic, anaerobic	---
	*E. coli* MG1655 [76]	450	Microaerobic	---
	*S. aureus* NCTC 8325 [72]	50	Microaerobic, aerobic	---
ALF186	*E. coli* MG1655 [76]	2000	Microaerobic	---
CORM-A1	*P. aeruginosa* [83]	10-500	Aerobic	Bacteriostatic
